# Direct and indirect estimation of adolescent sexual intercourse and contraceptive use in Rajasthan India: an application of the best friend methodology

**DOI:** 10.1186/s12905-024-03209-6

**Published:** 2024-06-27

**Authors:** Suzanne O. Bell, Danish Ahmad, Anoop Khanna, Haley L. Thomas, Caroline Moreau

**Affiliations:** 1 Department of Population Family and Reproductive Health, Johns Hopkins Bloomberg School of Public Health, 615 N. Wolfe Street, Baltimore, MD 21205 USA; 2 Indian Institute of Health Management Research, Rajasthan, India; 3 Soins et Santé Primaire, CESP Centre for Research in Epidemiology and Population Health U1018, Inserm, Villejuif, F-94805 France

**Keywords:** Adolescents, Reproductive health, Sexual behavior, Contraception, India, Surveys and questionnaires, Cross-sectional studies

## Abstract

**Background:**

Existing estimates of adolescent sexual and reproductive health (ASRH) behaviors may be a gross undercount given the sensitivity of this behavior in Indian culture. The objective of this study was to estimate ASRH behaviors in Rajasthan, India using direct questions and the best friend approach that seeks to reduce social desirability bias.

**Methods:**

We used population-based data of adolescents aged 15–19 in Rajasthan collected between September and December 2022. Data include whether the respondent and her closest female friend ever had a partner, ever had sex, ever used contraception, and were currently using contraception. We estimated respondent and best friend ASRH outcomes separately, overall and among unmarried adolescents for whom we anticipate social desirability bias is greatest.

**Results:**

The best friend approach performed well, with method assumptions largely met even before adjustments. Respondent and best friend estimates were similar among all adolescents except for current contraceptive use, which was higher for friends (though not significantly so). However, we observed large differences in ASRH behaviors between unmarried respondents and friends, with a significantly higher percentage of friends who ever had a partner (4.3% respondents, 11.6% friends), and a slightly higher percentage who ever had sex (2.4%, 3.8%) and who were currently using contraception (17.0%, 19.7% among those in need of contraception).

**Conclusions:**

We observed potential benefits of using the best friend methodology in estimating premarital sexual activity, but further work is needed to refine social network-based measures of sensitive adolescent behaviors in larger study samples to better understand ASRH needs.

## Background

Recent social and demographic shifts towards expanded education and delayed age at marriage are rapidly changing the social realities of adolescents in India, including their relational and sexual experiences. As the age at marriage increases, premarital relationships in adolescence and emerging adulthood are becoming more common despite stringent social norms proscribing sexual activity before marriage [1]. The growing dissonance between social expectations and lived experiences creates new challenges in meeting adolescent’s sexual and reproductive health needs.

Lack of information coupled with social stigmatization of premarital sexual intercourse and other sexual behaviors in India [2] are significant barriers to accessing sexual and reproductive health (SRH) services, potentially contributing to high levels of unmet need for family planning among adolescents [3, 4]. The government launched a national comprehensive adolescent health strategy in 2005 to promote SRH by integrating facility and community-based contraceptive service models and demand generation activities [5]. This strategy faced challenges during implementation [6], including cultural resistance [7]. It was subsequently replaced by *Rashtriya Kishor Swasthya Karyakram* (National Adolescent Health Program) in 2014, which embeds SRH in the broader context of adolescent health [7]. Its implementation is underway, highlighting the need for quality data to monitor adolescent sexual and reproductive health (ASRH) progress in a rapidly changing social landscape.

According to the National Family Health Survey 2019-21, one-quarter of young women in Rajasthan aged 20–24 were married before their eighteenth birthday and only 3.7% had a child before they turned 19, thus the majority of adolescents in this setting seek to avoid pregnancy [8]. However, results from two recent population surveys conducted in the state in 2018 and 2020 reveal concerning trends among adolescent girls regarding rising knowledge gaps and stigma related to contraception [9]. There was also limited inferred contraceptive needs given low levels of reported premarital sexual activity, but such results may misrepresent the extent of this behavior given the sensitivity surrounding it in Indian culture and the incentive to underreport [9–11] related to fears of humiliation (name calling) or loss of respect [9]. Direct estimates from 2018 suggest that only 19% of adolescents ages 15–19 in Rajasthan have ever had sex, this proportion dropping to 8% among 15–17-year-olds [2]. Yet another study revealed substantial underreporting of premarital sexual activity on a survey conducted in six Indian states, showing that 27% of young women 15 to 24 did not report sexual activity during a face-to-face interview but did so using the sealed envelope approach, an anonymized method intended to reduce social desirability bias [12]. A few studies conducted in slum areas indicated higher levels of premarital sexual intercourse among adolescents in India, but these finding may still underrepresent the extent of this behavior [13]. Other research on related ASRH outcomes in India reveal significant underreporting in standard survey approaches, including studies on abortion [14], masturbation, same-sex relations, and condom use [11]. Such underestimates hinder the government’s capacity to project and appropriately address ASRH needs.

To complement direct estimations of key ASRH indicators, we sought to apply the best friend method, a promising methodology to address social desirability bias in the reporting of sensitive SRH behaviors. This method consists of creating a surrogate representative sample based on respondents’ social networks and estimating the sensitive behavior in the surrogate population [15, 16]. Though results have been mixed, the approach has successfully been used to improve estimates of abortion incidence across several geographies in sub-Saharan Africa and Southeast Asia, generally producing higher estimates than direct reporting [14–21]. This approach has also been used to measure the extent of people having multiple sexual partners more accurately [16]. The implementation of this methodology to estimate abortion in Rajasthan yielded promising results, increasing annual abortion incidence estimates from 9.5 per 1,000 women of reproductive age based on self-reports to 23.0 per 1,000 women [14]. Thus, we aimed to apply this social network-based approach to better estimate sexual intercourse and contraceptive use among adolescents aged 15–19 in Rajasthan, India. There are different applications of this indirect approach, depending on the number of friends and relationship criteria considered, but all share common assumptions: (1) the surrogate friend sample is representative of the original population, (2) the respondents know about friends’ behavior, (3) respondents are more willing to report on their friends’ sensitive behaviors than their own (reduced social desirability bias) [22–26].

The objective of this study was to estimate ASRH behaviors using direct and best friend approaches, adjusting for violations of the social network-based method assumptions. Specifically, we sought to estimate the percent of adolescents who have ever been in a relationship or been sexually active and the percent of current contraceptive use among sexually active adolescents. We examined these behaviors among all adolescents and unmarried adolescents specifically, as we anticipated the social desirability bias would be higher for unmarried adolescents.

## Methods

### Data

We used data from the Performance Monitoring for Action (PMA) study [2] conducted in Rajasthan between September and December 2022. The PMA Rajasthan panel employed an urban/rural stratified two-stage sampling approach with probability proportional to size sampling of clusters within each stratum to identify a representative sample of women aged 15–49. In total, 134 clusters from 33 districts were selected in 2020 (Phase 1), and 35 households within each selected cluster were randomly included. All women aged 15–49 living in the selected households were invited to participate, comprising a representative sample of 5,465 reproductive aged women (response rate 98.1%). A total of 5,114 (93.6%) of these women agreed to be re-interviewed. In Phase 3, conducted in 2022, a total of 5,481 females were surveyed, including 4,696 women from Phase 1 or 2 and 785 (14.3%) women from replacement households randomly selected from the sampling frame to replace those lost to follow-up; replacement sampling produces representative cross-sectional estimates [9]. Our analytic population included 904 adolescents (15–19) who were part of the cross-sectional sample (i.e., panel women and replacement households) and completed the interview. All adolescents provided informed written and verbal consent prior to participation in accordance with approved Institutional Review Board procedures. Ethical approval for this study design was provided by the Indian Institute of Health Management Research (IIHMR) Institutional Review Board for Protection of Human Subjects in Rajasthan and the Bloomberg School of Public Health at Johns Hopkins University.

Consenting adolescents participated in a 45-minute interview conducted by trained resident interviewers. The survey, administered by interviewers face-to-face in Hindi, solicited information on respondents’ sociodemographic background, partnerships, whether they ever had sexual intercourse, births, fertility intentions, and contraceptive use (specifically whether the woman or her partner were currently doing something or using any method to delay or avoid getting pregnant). The survey also included a module inquiring about the respondent’s closest female friend aged 15 to 19, including the friend’s sociodemographic background, partnership status, whether ever had sexual intercourse, and current contraceptive use (analogous to the respondent question).

### Measures

The primary outcomes include lifetime partnership and whether they ever had sexual intercourse among all adolescents and current contraceptive use among sexually active adolescents who were not pregnant or seeking a pregnancy at the time of the survey. We examined these outcomes overall and among unmarried adolescents in both the respondent sample and the friend surrogate sample.

### Statistical analysis

The analysis started with a description of the respondent sample and an exploration of the potential selection bias of the surrogate sample of friends. We compared the sociodemographic characteristics of respondents who reported having 0 friends to those who reported 1 or more friends to estimate the impact of missing friends, as respondents with no friends contributed no information to the surrogate sample. Sociodemographic characteristics examined included age (15–17, 18–19), education (never, primary, secondary, higher), marital status (married, not married), currently working (yes, no), religion of household (Hindu, Muslim, other), wealth (tertile based on household assets, building materials water, and sanitation), residence (urban, rural), and number of children (0, 1+). We also examined differences in respondent and friend sociodemographic characteristics (except for religion and wealth, which we did not collect for friends). For both sets of comparisons, we used design-based F-tests to evaluate whether differences were statistically significant.

We made a number of adjustments to account for observed biases in the friend sample. To account for “missing” friends with no social network, corresponding to the respondents who reported having no friends, we incorporated this sub-population of respondents into the friend sample to create an adjusted surrogate sample. Given concerns about underreporting of sexual intercourse in the self-reported data, we used a Poisson model to predict the likelihood of these “missing” friends having ever had sexual intercourse by regressing the respondents’ socioeconomic characteristics on the corresponding friend sexual intercourse data. We also separately used a similar model to predict the likelihood of these “missing” friends ever having a partner and currently using contraception. To further improve the representativeness of the surrogate sample, we constructed post-stratification weights for the surrogate sample to try to replicate the sociodemographic distribution of the respondent sample. Finally, to adjust for incomplete knowledge of friend’s sexual activity, which we viewed as most sensitive, we calculated a transmission bias adjustment factor as one divided by the proportion of respondents who had ever had sex who had told their friend about being sexually active.

We used design-based F-tests to evaluate whether respondent and adjusted friend characteristics were statistically different and used the adjusted friend data to estimate our ASRH indicators. We then estimated the prevalence of each outcome – ever had a partner, ever had sex, and currently using contraception – for respondents and friends, using both the unadjusted and adjusted friend data to show the impact of our adjustments for potential method assumption violations. While the percent who ever had a partner or had sex was estimated among the entire sample, we examined current contraceptive use among those who were not currently pregnant and did not wish to become pregnant in the next year. We conducted the same analyses among the subset of unmarried respondents and friends, for whom we anticipated greater benefit of the best friend methodology due to the sensitivity of premarital sexual activity. Lastly, we examined the correlates of having ever had sex or currently using a contraceptive method – two particularly salient ASRH outcomes – among the respondent and adjusted friend sample. We conducted the same analysis among unmarried adolescents in relation to sexual intercourse but were unable to do so for current contraceptive use given the small sample size.

All analyses were conducted in Stata version 15.1 (College Station, TX). Given the complex sampling design, we applied survey-design weights and calculated standard errors using the Taylor linearization method to account for clustering.

## Results

Among the total respondent sample of 904 female adolescents aged 15–19 years, 766 (87.6%) reported having at least one close female adolescent friend. Adolescents who reported no friends were less educated and more likely to live in the poorest households compared to those who reported at least one friend (Table 1).

**Table 1:** Characteristics of female respondents aged 15 to 19 overall and by number friends reported in Rajasthan, India*

		All respondents		0 Friends		≥ 1 Friend	
		%	*N*	%	*N*	%	*N*
Age							
	15–17	63.6	562	63.2	86	63.6	476
	18–19	36.4	342	36.8	52	36.4	290
Education							
	Never/Primary	14.4	154	**27.5**	**37**	**12.5**	**117**
	Secondary	32.5	287	**29.9**	**43**	**32.8**	**244**
	Higher	34.8	303	**31.2**	**39**	**35.2**	**264**
	Tertiary	18.4	160	**11.3**	**19**	**19.4**	**141**
Currently married							
	No	86.4	761	85.0	117	86.6	644
	Yes	13.6	143	15.0	21	13.4	122
Currently working							
	No	83.5	733	76.6	104	84.5	629
	Yes	16.5	169	23.4	33	15.5	136
Religion of household							
	Hindu	85.1	746	72.4	106	86.9	640
	Muslim	13.9	135	25.3	29	12.2	106
	Other	1.1	15	2.3	3	0.9	12
Wealth tertile							
	Poorest	31.8	321	**49.8**	70	**29.2**	251
	Middle wealth	36.6	334	**27.9**	44	**37.9**	290
	Wealthiest	31.6	249	**22.4**	24	**32.9**	225
Residence							
	Rural	79.1	652	70.7	98	80.3	554
	Urban	20.9	252	29.3	40	19.7	212
Parity							
	0	96.9	867	97.3	131	96.9	736
	1+	3.1	31	2.7	3	3.1	28
Ever had sex							
	No	86.6	761	87.8	121	86.5	640
	Yes	13.4	143	12.2	17	13.5	126
Ever used family planning							
	No	97.1	876	98.7	136	96.5	736
	Yes	2.9	28	1.3	2	3.5	30
Currently using contraception							
	No	97.4	878	98.7	136	97.2	742
	Yes	2.6	26	1.3	2	2.8	24
Total		100.0	904	100.0	138	100.0	766

*Estimates weighted; bold indicates *p*-value for design-based F-test comparing respondents with 0 to 1 + friends less than 0.05

Table 2 shows the demographic characteristics of the respondents and the unadjusted and adjusted characteristics of their closest female friends. While the distribution of education was higher among the unadjusted friend sample compared to respondents, all characteristics were similar when comparing the adjusted surrogate sample to the respondents. Approximately 60% of the sample was 15-17-year-olds (63.6% of respondents, 61.0% of friends), eight in ten lived in rural areas (79.1% of respondents and 79.0% of friends), more than half had more than a secondary level of education (53.2% of respondents and 51.7% of friends), and approximately 1 in 6 were currently working (16.5% of respondents, 16.0% of friends). A minority of adolescents were married (13.6% respondents versus 12.2% of friends) or had any children (3.1% of respondents, 2.3% of friends). Among respondents who reported being sexually active and having a close female friend, 80.3% told their friend they had had sex. Thus, the transmission bias adjustment factor for sexual intercourse was 1.25 (1/0.803), which we applied to the adjusted surrogate sample to calculate the adjusted estimate of the prevalence of friends who had ever had sexual intercourse.

**Table 2:** Characteristics of female respondents aged 15 to 19 and their closest female friends aged 15 to 19 in Rajasthan, India*

		Respondent		Unadjusted friend		Adjusted friend**	
		%	*N*	%	*N*	%	*N*
Age							
	15–17	63.6	562	61.8	478	61.5	564
	18–19	36.4	342	38.2	286	38.5	340
Education							
	Never/Primary	14.4	154	**10.0**	**103**	12.8	140
	Secondary	32.5	287	**36.6**	**262**	34.5	305
	Higher	34.8	303	**34.3**	**270**	34.0	309
	Tertiary	18.4	160	**19.1**	**131**	18.8	150
Currently married							
	No	86.4	761	90.3	679	87.8	797
	Yes	13.6	143	9.7	86	12.2	107
Currently working							
	No	83.5	733	87.0	658	83.9	766
	Yes	16.5	169	13.0	104	16.1	137
Residence							
	Rural	79.1	652	80.2	554	78.6	652
	Urban	20.9	252	19.8	212	21.4	252
Parity							
	0	96.9	867	98.3	749	97.6	880
	1+	3.1	31	1.7	17	2.4	20
Total		100.0	904	100.0	766	100.0	904

*Estimates weighted, Ns unweighted; bold indicates *p*-value for design-based F-test (reference respondents) less than 0.05

**Estimates include respondent characteristics in place of “missing” friends; post-stratification weights applied

### ASRH indicators

Less than 1 in 5 adolescents self-reported ever being in a relationship (17.3%), and fewer self-reported ever having had sex (13.4%) (Table 3). Among respondents who had ever had sex and were not pregnant or trying to become pregnant, 29.0% indicated they were currently using contraception (Table 3). Adjusted friend estimates showed slightly higher reports of partnerships (21.4%), similar estimates of sexual intercourse (12.7%), and higher current contraceptive use (37.0%), though none of the differences were statistically significant. However, differences between friends and respondents were greater among unmarried adolescents, showing a statistically significant increase from 4.3% of unmarried respondents ever being in relationship to 11.6% based on adjusted unmarried friend data (Table 3). Friends’ estimates of premarital sexual intercourse were also higher than those of respondents, although they remained generally low even after adjustment for transmission bias (3.8% of friends versus 2.4% of respondents). Contraceptive use among unmarried friends who had ever had sex but were not pregnant and not trying to conceive was similarly only somewhat higher than that of unmarried respondents who had ever had sex, but not significantly so (19.7% of friends versus 17.0% of respondents).

**Table 3:** Characteristics of female respondents age 15 to 19 and their closest female friends age 15 to 19 in Rajasthan, India*

		All				Unmarried			
		Respondent		Friend**		Respondent		Friend**	
		%	SE	%	SE	%	SE	%	SE
Ever had a partner		*n* = 904		*n* = 899		*n* = 761		*n* = 793	
	Unadjusted	17.3	2.6	19.4	3.6	4.3	1.5	**10.8**	**2.8**
	Adjusted	--	--	21.4	3.3	--	--	**11.6**	**2.5**
Ever had sex		*n* = 904		*n* = 899		*n* = 761		*n* = 793	
	Unadjusted	13.4	2.4	**8.5**	**1.8**	2.4	0.9	2.4	1.1
	Adjusted	--	--	12.7	2.2	--	--	3.8	1.3
Currently using family planning		*n* = 98		*n* = 68		*n* = 28		*n* = 28	
	Unadjusted	29.0	5.9	40.4	9.4	17.0	5.5	21.5	1.6
	Adjusted	--	--	37.0	9.1	--	--	19.7	1.1

*Estimates weighted, Ns unweighted; bold indicates *p*-value for design-based F-test (reference respondents) less than 0.05

**Adjusted estimates include respondent characteristics in place of “missing” friends and post-stratification weights (transmission bias adjustment factor also applied to estimates of prior sex). Adjusted friend n does not equal 904 due to missing respondent data on number of children, which informed the Poisson model

***Unadjusted friend Ns for ever had a partner, ever had sex, and currently using family planning analyses are 766, 766, and 59, respectively, for all friends and 679, 679, and 25, respectively for unmarried friends

Patterns of sexual intercourse among respondents differed (though not significantly) by age, marital status, education, and wealth, with increased reporting among married, older, less educated, and less wealthy adolescents (Table 4). Similar patterns were observed among friends. Among sexually active respondents seeking to avoid pregnancy, we found greater use of contraception among older, married, and rural adolescents, with less use among those with the most education and wealth. While sample size limits interpretation of friend findings, we note higher reporting of contraceptive use among friends with higher and tertiary education and those residing in urban areas compared to their respondent counterparts. Among unmarried adolescents, differences in estimates of sexual intercourse were consistently slightly higher between respondents and friends but not significantly so.

**Table 4:** Sexual and reproductive health behaviors among respondents and friends aged 15 to 19 overall and by background characteristics*

		All adolescents								Unmarried adolescents			
		Ever had sex				Currently using contraception				Ever had sex			
		Respondent		Adjusted friend**		Respondent		Adjusted friend**		Respondent		Adjusted friend**	
		%	*N*	%	*N*	%	*N*	%	*N*	%	*N*	%	*N*
Age													
	15–17	6.2	562	6.9	564	17.8	38	34.8	32	2.2	511	3.4	526
	18–19	25.9	342	21.9	340	35.3	60	38.3	36	2.7	250	4.7	267
Education													
	Never/Primary	29.2	154	30.9	140	35.6	30	29.7	18	3.9	112	7.2	104
	Secondary	14.2	287	**9.7**	**305**	48.4	29	65.3	17	1.2	233	2.2	273
	Higher	9.4	303	11.4	309	9.5	26	28.1	25	3.2	271	4.6	277
	Tertiary	7.2	160	8.0	150	2.0	13	26.2	8	1.9	145	3.6	139
Currently married													
	No	2.4	761	3.8	797	17.0	28	19.7	28	n/a	n/a	n/a	n/a
	Yes	83.2	143	**76.1**	**107**	32.5	70	45.3	40	n/a	n/a	n/a	n/a
Wealth tertile													
	Poorest	16.6	321	--	--	21.8	44	--	--	4.5	252	--	--
	Middle wealth	18.4	334	--	--	37.2	45	--	--	2.0	278	--	--
	Wealthiest	4.3	249	--	--	5.8	9	--	--	0.9	231	--	--
Residence													
	Rural	14.3	652	13.0	652	30.8	87	32.2	58	2.9	532	4.1	560
	Urban	9.9	252	11.4	252	16.0	11	59.4	10	0.7	229	2.7	233
Parity													
	0	10.7	867	10.9	880	21.6	71	35.7	55	--	--	--	--
	1+	100.0	31	83.6	20	46.6	27	41.7	13	--	--	--	--
Total		13.4	904	12.7	899	29.0	98	37.0	68	2.4	761	3.8	793

*Estimates weighted, Ns unweighted; bold indicates *p*-value for design-based F-test (reference respondents) less than 0.05

**Estimate include respondent characteristics in place of “missing” friends; post-stratification weights applied

## Discussion

This study sought to apply a social network-based approach to improve ASRH estimates by reducing potential social desirability bias related to premarital relationships, sexual intercourse, and contraceptive behaviors among adolescent girls in Rajasthan, India under the assumption that current direct reports are underestimates. The findings are mixed, as overall indirect estimates of sexual intercourse and contraceptive use were not significantly higher compared to direct estimates, while estimates for unmarried adolescents were significantly higher in relation to ever having a partner, but were not statistically significantly higher in relation to estimates of sexual intercourse and current contraceptive use. Social patterns of sexual intercourse and contraceptive use were generally comparable between the two methodologies.

Overall, few adolescents aged 15 to 19 had ever had sexual intercourse, regardless of the estimation approach, and sexual intercourse mainly occurred in the context of marriage. However, among unmarried adolescents, a larger proportion had ever engaged in a relationship (4.3% of respondents and 11.6% of friends) relative to the less commonly reported sexual intercourse (2.4% of respondents and 3.8% of friends), suggesting an extended time of non-sexual premarital romance in committed or non-committed partnerships. These findings support results from prior research [27], including studies across India showing greater romantic interest than sexual encounters among unmarried adolescent girls [1, 28, 29], although a few studies conducted in slum areas report higher levels of premarital sex than those in the broader population [13].

Our low levels of premarital sex from self-reports are perhaps an underestimate, with friend estimates of 3.8% compared to 2.4% for respondents, though we were not powered to detect differences of this magnitude. However, even indirect estimates remain low, which may be due to violations of the social network-based method assumptions or reflect the reality of girls’ sexual transitions into adulthood in this socially prescriptive environment. Our surrogate sample may have been skewed due to 12.4% of respondents with no social relations who were essentially “imputed” by including the respondents with no friends in the adjusted surrogate sample. However, there were few differences between adolescents with or without friends, and the distribution of friends’ sociodemographic characteristics generally matched that of respondents, suggesting limited selection bias of the surrogate sample. Respondent’s knowledge about their friends’ behaviors (transmission bias) has been a more serious concern in application of this methodology to measurement of abortion [20, 21, 30], as the social network-based approach assumes respondents know about the sensitive behaviors of their friends. The proportion of respondents who share their abortion experience with their friends varies by social context, depending on how much women may rely on their social network to access abortion care in restrictive legal settings [31, 32]. These conditions don’t apply to sexual intercourse, which doesn’t require help from one’s social network to be performed, however, the social context and significant social restrictions on premarital adolescent sex likely influence sharing between friends. In this study, nearly 80% who reported prior sexual intercourse indicated they had told their friend about this experience; thus, the transmission bias adjustment was relatively small (1.25). However, this adjustment assumes the sharing patterns of respondents who do and do not self-report prior sexual intercourse, and that the level of sharing between friend dyads is the same in both directions. Additionally, due to the small number of unmarried respondents who reported having had sex and who had a close female friend (*n* = 11) we could not calculate this adjustment factor separately for unmarried adolescents.

While our estimates of premarital SRH behaviors may be low, they may also reflect a singular pattern of non-sexual partnerships in India, especially among adolescent girls who have internalized social scripts ostracizing adolescent sexuality [1, 33]. These scripts also prevent adolescents who need contraception from accessing SRH services for fear of social sanctions. In our study, between 63% (friends) and 71% (respondents) of sexually active adolescents wishing to prevent a pregnancy were not using contraception. Findings from the 2018 PMA Rajasthan survey show 40% of girls aged 15–19 agreed with the statement “adolescents using contraception are seen as promiscuous,” which may constrain their willingness to access family planning services or share their contraceptive needs with providers, exposing them to the risk of unintended pregnancy [2]. With increasing duration of premarital romantic relationships, it is critical to address these social barriers, not only through sexual education among adolescents, but through wider community engagement to create an enabling environment for adolescents to experience healthy and safe relationships.

Beyond marital status, findings suggest social patterns of sexual intercourse were evident, with earlier sexual debut among less educated and poorer adolescents (though results were not statistically significant). These patterns mirror national patterns of age at first sex, which increases with education [8]. Despite expanded education among girls in India, they receive less than four years of formal education, on average, with 40% dropping out before their fifth year of school, limiting their access to school-based sexual education [34]. These findings underscore the importance of early interventions to dispense age-appropriate comprehensive sexual education throughout the educational curriculum, reaching adolescents before they drop out of school and with continued community outreach to complement school programs for out of school adolescents.

Our results need to be interpreted with several limitations in mind. First, the low prevalence of sexual intercourse reduces our sample size for estimating contraceptive behaviors among sexually active adolescents, leading to substantial imprecision of estimates of contraceptive behaviors. Likewise, analysis of intercourse among unmarried adolescents is limited given the small number of adolescents in this group of both respondents and friends. In this respect, the indirect methodology made little difference given small improvements in estimates of ASRH indicators. As previously discussed, if the adjustment for transmission bias does not accurately account for incomplete visibility of these ASRH behaviors, the adjusted friend estimates is biased. Lastly, this study does not capture the ASRH experiences or needs of boys and non-binary populations in this context and we did not collect information on sexual orientation, limiting the generalizability of our findings.

Despite these limitations, we still believe this study contributes to expanding our methodological quest for addressing quality of ASRH measurement. We found slight benefits of the best friend methodology in estimating ASRH indicators in Rajasthan, particularly with regard to premarital relationships, and suggest further qualitative and quantitative work to refine social network-based measures of sensitive adolescent behaviors to better understand ASRH needs. We also draw attention to the increasing SRH needs of unmarried adolescents who spend more time in non-sexual premarital partnerships, but as reported in prior PMA results, lack the knowledge, agency and social support to make autonomous decisions about marriage, sexual debut and contraceptive use [2, 9].

## Conclusions

We used the best friend methodology to estimate sensitive ASRH behaviors using population-based data of adolescents aged 15–19 in Rajasthan, India. We observed potential benefits of using the best friend methodology in estimating sexual activity among unmarried adolescents, among which we hypothesized social desirability bias would be the greatest. However, further work is needed to refine social network-based measures of sensitive adolescent behaviors in larger study samples to better understand ASRH needs, which remind difficult to address given potential underreporting. As premarital romantic relationships become more common among youth in India, it is essential we have accurate information on the extent and nature of sexual encounters among this population to inform policies and programs that seek to ensure adequate sexual education and ASRH services.

## Data Availability

The data that support the findings of this study are available from Performance Monitoring for Action, and access to the data may be granted upon reasonable request (10.34976/zwsv-md35).

## Abbreviations

ASRH: Adolescent sexual and reproductive health
EAs: Enumeration areas
IIHMR: Indian Institute of Health Management Research
PMA: Performance Monitoring for Action
SRH: Sexual and reproductive health
